# Circulating tumor cells in advanced non-small cell lung cancer patients are associated with worse tumor response to checkpoint inhibitors

**DOI:** 10.1186/s40425-019-0649-2

**Published:** 2019-07-10

**Authors:** Menno Tamminga, Sanne de Wit, T. Jeroen N. Hiltermann, Wim Timens, Ed Schuuring, Leon W. M. M. Terstappen, Harry J. M. Groen

**Affiliations:** 1 Department of Pulmonary Diseases, University of Groningen, University Medical Center Groningen, Hanzeplein 1, P.O. Box 30.0001, 9700 RB Groningen, The Netherlands; 2 Department of Medical Cell BioPhysics, Faculty of Sciences and Technology, University of Twente, Enschede, The Netherlands; 3 Department of Pathology and Medical Biology, University of Groningen, University Medical Center Groningen, Groningen, The Netherlands

**Keywords:** Circulating tumor cells (CTC), Non-small cell lung cancer (NSCLC), Immunotherapy, Checkpoint inhibitors, Tumor derived extracellular vesicles (tdEV), Durable response, Liquid biopsy

## Abstract

**Background:**

Non-small cell lung cancer (NSCLC) patients treated with checkpoint inhibitors show long lasting responses, but it is hard to predict which patients will profit from this treatment with the currently used marker, programmed death ligand 1 (PD-L1). We hypothesized that circulating tumor cells (CTC) or tumor derived extracellular vesicles (tdEV) are markers of treatment efficacy.

**Methods:**

Patients with advanced NSCLC treated with checkpoint inhibitors were included. Blood was drawn at baseline (T0) and at 4 weeks of treatment (T1) for analysis of CTC and tdEV using CellSearch®. Tumor response was classified as partial or complete response based on the response evaluation criteria in solid tumors (RECISTv1.1) measured 4–6 weeks after start of treatment. Durable response was defined as stable disease, partial or complete response without disease progression at 6 months. Analyses were adjusted for covariables including PD-L1 expression.

**Results:**

We included 104 patients (30 with a tumor response, 74 non-responders, 2 responses not evaluable due to early death); 63 patients provided T1 samples. All patients were treated with PD-L1 inhibitors. The majority of patients received second (85%) or third line (treatment with nivolumab monotherapy (89%).

CTC were present in 33/104 patients at T0 (32%) and 17/63 at T1 (27%), 9/63 patients had CTC (14%) at both time points. The presence of CTC, both at T0 (OR = 0.28, *p* = 0.02,) and T1 (OR = 0.07, *p* < 0.01), was an independent predictive factor for a lack of durable response and was associated with worse progression free and overall survival. More tdEV were associated with shorter survival but not with response rate.

**Conclusion:**

CTC occur in one third of advanced NSCLC patients and their presence is a predictive factor for a worse durable response rate to checkpoint inhibitors. tdEV are associated with shorter survival but not with response.

**Electronic supplementary material:**

The online version of this article (10.1186/s40425-019-0649-2) contains supplementary material, which is available to authorized users.

## Introduction

Lung cancer accounts for 13% of newly diagnosed cancer cases and is responsible for 19% of cancer related deaths, translating to over a million deaths worldwide annually [1, 2]. While checkpoint inhibitors have been able to ensure long-lasting survival, this is only achieved in approximately 20% of non-small cell lung cancer (NSCLC) patients, whereas the remainder experiences little or no benefit from this treatment [3, 4]. Some patients have responses that are remarkable durable (> 6 months), but these are a subset of the patients who have an initial response. Imaging does not identify these patients, as even patients with stable disease can remain stable for a prolonged time.

A biomarker that can accurately predict the response to checkpoint inhibitors would therefore be of great clinical benefit. At present the expression of programmed death-ligand 1 (PD-L1) measured by immunohistochemistry (IHC) on tumor biopsies predicts tumor response to a certain extent, but is not a robust predictor for an individual patient [5].

Possible early markers of response to checkpoint inhibitors are circulating tumor cells (CTC) and tumor-derived extracellular vesicles (tdEV) [6–9]. Both are derived directly from the original tumor or metastatic sites. CTC are epithelial tumor cells that have been expunged into the bloodstream and can settle at a secondary site to form metastases. Their presence has been reported to be an independent prognostic marker of relative short overall survival (OS) in several types of cancer, including NSCLC [10–16]. It is possible that the presence of CTC is a reflection of the tumor burden or invasiveness causing them to be associated with worse survival [10, 13, 17]. These characteristics allow them to be used as a liquid biopsy in a less invasive approach to obtain information on prognosis and treatment management.

Similar to CTC, tdEV are derived from the tumor and associated with worse survival in NSCLC and hormone refractory prostate cancer [8, 18]. They are vesicles expressing epithelial cell adhesion molecule (EpCAM) and cytokeratin, but in contrast to CTC, do not have a nucleus. Recently, de Wit et al. showed that tdEV can be found in NSCLC and are associated with survival, using tdEV≥18 per 7.5 mL blood as a cut off, based on healthy controls [8].

Considering their value as a prognostic marker, we hypothesized that the presence of CTC and higher tdEV counts (≥18 tdEV /7.5 mL) are associated with a worse early and durable tumor response rate to checkpoint inhibitors in advanced NSCLC patients. For this purpose we determined CTC and tdEV counts in a prospective exploratory cohort of real life NSCLC patients treated with checkpoint inhibitors.

## Methods

### Patients

Patients with advanced NSCLC (stage IIIB and IV), eligible for treatment with checkpoint inhibitors towards PD-L1 or PD − 1 receptors were asked to participate in this prospective exploratory cohort study. Patients received routine checkpoint inhibitors intravenously. Blood samples were drawn in the week before the start of checkpoint inhibitor therapy (T0) and four to 6 weeks after start of therapy (T1). All assessments were performed by the treating physician and occurred without knowledge of CTC and tdEV counts. Measured variables included: age; gender; Eastern Cooperative Oncology Group Performance Score (PS); smoking status; stage; histology; treatment lines; tumor size; number of locations of metastases; PD-L1 expression detected with the 22C3 antibody; checkpoint inhibitor medication; tumor response, time to progression and overall survival.

Additionally for adenocarcinoma patients tumor DNA mutations were detected by next generation sequencing with the Ion Torrent using an in-house panel (IonPGM-v002) targeting hotspots in 24 genes with 82 amplicons (targeted genes are: AKT, ALK, BRAF, EGFR, ERBB2, ESR1, GNA11, GNAQ, GNAS, H3F3B, HRAS, IDH1, JAK2, KIT, KRAS, MAP2K1, MET, NRAS, PDGFRA, PIK3CA, POLE and ROS1), while FISH was used to detect rearrangements of the ALK, ROS1 and RET genes (Vysis Break Apart FISH probes). ALK rearrangements were confirmed with immunohistochemistry.

For squamous cell carcinoma patients amplifications of FGFR1 were detected with FISH [19, 20].

The study was approved by the Medical Ethical Committee and informed consent was obtained from all patients (METc nr. 2017/217).

### Tumor response

Early tumor response was measured 4–6 weeks after start of treatment using the revised Response Evaluation Criteria In Solid Tumors (RECIST) v1.1 [21]. Patients with stable disease (SD), progressive disease (PD) and patients who had a non-evaluable response (NE) due to early death were deemed as having no early tumor response, while patients with a partial response (PR) or complete response (CR) were seen as responders.

Durable response was defined as patients who had either SD, PR or CR, with no progression measured by RECIST v1.1 for at least 6 months [3, 4].

### Enumeration of EpCAM^high^ CTC and tdEV with CellSearch

Aliquots of 7.5 mL whole blood were enumerated for CTC and tdEV with the CellSearch® Circulating Tumor Cell Kit within 48 h after blood draw in a CellSave tube (Menarini Silicon Biosystems, Huntingdon Valley PA, USA). Blood from the CellSave tube was transferred to a CellSearch conical tube and centrifuged for 10 min at 800 g without using the brake, after which the sample was placed in the CellSearch Autoprep for analysis. The blood samples were immunomagnetically enriched for cells and tdEVs expressing EpCAM and stained with DAPI, CK-PE and CD45-APC. Image acquisition of the CellSearch cartridges containing the enriched and stained cell suspension was performed on the CellTracks Analyzer II.

### Scoring of CTC and tdEV

CTC candidates in the images from the cartridges were identified by the CellTracks Analyzer II and presented to a trained operator for CTC classification per manufacturer instructions. CTC were defined as objects larger than 4 μm in diameter, stained with DAPI and CK, lacked CD45 staining and had morphological features consistent with that of a cell [22]. All CellTracks images from all cartridges were analyzed using the open source imaging program ACCEPT [23–25]. In short, the ACCEPT toolbox uses an advanced multi-scale segmentation approach and extracts fluorescence intensity and shape measurements for every event found. Based on selection criteria selected by the user, the program can present all events conforming to the criteria. The selection criteria used for tdEV were: CK mean intensity ≥60, CK maximum intensity ≥90, CK standard deviation of intensity ≥0.15, CK size < 150 μm^2^, CK perimeter ≥3.2 μm (≥5 pixels), CK roundness < 0.80 (where 0 is perfectly round and 1 is a perfect line), CK perimeter to area <  1.1, DNA mean intensity < 5, CD45 mean intensity < 5. As the blood was centrifuged at 800 g and the plasma discarded before processing on the CellSearch system, the detected tdEV were relatively large vesicles (> 1 μm) [26].

### Statistical analysis

Descriptive statistics were used for clinical characteristics. Patients were separated in favorable and unfavorable groups based on the presence of CTC, and for 18 or more tdEV. The cut off value of 18 tdEV was used previously by de Wit et al. and is based on the mean tdEV count in 35 healthy donors (tdEV = 5.1), with two standard deviations (6.7) [8].

The change in CTC and tdEV over time was calculated. This variable was subsequently dichotomized into patients with 0 CTC at both time points or decreasing CTC/tdEV counts (favorable group) and patients with increasing CTC or tdEV counts (unfavorable).

Differences between patients in the favorable and unfavorable group were compared by means of T-tests and Mann-Whitney U tests for continuous variables and Χ^2^ tests or Fishers exact test for categorical variables.

The primary endpoints were differences in early tumor and durable response rates between patients with and without CTC and elevated or not elevated tdEV. If the Χ^2^ or Fishers Exact test were significant, binary logistic regression was used corrected for clinical parameters and expressed as odds ratios (OR) for response (> 1 indicates response benefit). In this multivariable model, covariables were selected in a backward conditional method, with *p* = 0.1 as a cutoff. In short, all previously mentioned variables were included in the base model. Covariables with a *p* > 0.1 were one by one removed from the model, starting with the highest *p* value, until all variables in the model had *p* < 0.01. The covariables in the final model are reported.

Secondary endpoints, PFS and OS, were investigated using Cox regression analyses. Covariables for these multivariable models were selected in the same way as for the logistic regression analyses. Corrected Hazard ratios (> 1 indicates shorter survival) and *p*-values were provided for CTC and tdEV independently. In all analyses, a *p* value of 0.05 or smaller was deemed significant. Outcomes from the logistic regressions, indicating an association with response rates were deemed predictive, while associations from the Cox regression analyses, indicating an association with survival, were seen as prognostic.

## Results

A total of 104 patients with advanced NSCLC who started checkpoint inhibitors were included. T1 samples (obtained between four and six weeks after start treatment) were obtained in 63 of these cases. Of 41 patients no T1 sample was obtained: 24 had progression or deceased before the second sample could be taken, one patient refused a second sample and 16 cases could not be obtained or processed. Mutations were detected in 47/104 patients (45%), mostly KRAS mutations (*n* = 33/104; 32%). These mutations were not significantly associated with tumor response.

Early tumor responses (PR or CR measured at 4–6 weeks by RECISTv1.1) were observed in 30/104 patients (29%), with 4 CR, 26 PR, 24 SD and 48 PD. Two patients had a non-evaluable response due to early death (denoted as PD). Durable responses (SD, PR or CR measured at 6 months) were observed in 40/104 patients (38%).

Patient characteristics are described in Table 1, with an overview of CTC and tdEV counts in Table 2.

**Table 1:** Characteristics of advanced NSCLC patients treated with checkpoint inhibitors

	Total population (*n* = 104)	Patients with CTC at T0 (*n* = 33)	Patients without CTC at T0 (*n* = 71)
	n (%)	n (%)	n (%)
Age			
Median (range)	65 (29–83)	67 (41–83)	65 (29–80)
Gender			
Male	58 (44)	17 (51)	41 (58)
Female	46 (56)	16 (49)	30 (42)
ECOG PS*			
0	50 (48)	9 (27)	41 (58)
1	52 (50)	23 (70)	29 (41)
2	2 (2)	1 (3)	1 (1)
Smoking status			
Smokers	94 (90)	28 (85)	66 (93)
Non smokers	3 (3)	2 (6)	1 (1)
Unknown	7 (7)	3 (9)	4 (6)
Stage			
III	12 (11)	1 (3)	1 (16)
IV	92 (89)	32 (97)	60 (84)
Histology			
Adenocarcinoma	76 (73)	24 (72)	52 (73)
Squamous cell carcinoma	27 (26)	8 (24)	19 (27)
Carcinosarcoma	1 (1)	1 (4)	0 (0)
Therapy line			
1	4 (4)	3 (4)	1 (3)
2	87 (84)	59 (83)	28 (85)
≥3	13 (12)	9 (13)	4 (12)
Metastatic sites			
0	15 (14)	2 (6)	13 (18)
1	37 (36)	13 (41)	24 (34)
2	35 (34)	12 (38)	23 (32)
3	10 (10)	4 (13)	6 (9)
> 3	6 (6)	1 (3)	5 (7)
Mutations ^a^			
None identified	46 (44)	18 (55)	39 (55)
KRAS	33 (32)	9 (27)	24 (34)
Other	14 (13)	6 (18)	8 (11)
PD-L1 ^b^			
< 1%	44 (43)	16 (49)	28 (39)
1–49% expression	19 (18)	7 (21)	12 (17)
≥50% expression	18 (17)	5 (15)	13 (18)
Not evaluable ^c^	23 (22)	5 (15)	18 (25)
Therapy			
Nivolumab	89 (85)	29 (85)	60 (83)
Pembrolizumab	8 (8)	2 (6)	6 (9)
Atezolizumab	5 (5)	1 (3)	4 (7)
Ipilimumab/Nivolumab	2 (2)	1 (3)	1 (1)
Response ^d^			
Complete Response	4 (4)	0 (0)	4 (6)
Partial Response	26 (25)	7 (21)	19 (27)
Stable Disease	24 (23)	5 (15)	19 (27)
Progressive Disease	50 (48)	21 (61)	29 (39)
Durable response ^e^			
> 6 months	64 (62)	7 (21)	33 (46)
< 6 months	40 (38)	26 (79)	38 (54)

*Eastern Cooperative Oncology Group Performance Score, patients with CTC had significantly more often PS ≥1 than patients without CTC (*p* = 0.02)

^a^Mutations were identified by NGS, specifically the Ion Torrent using an in-house panel (IonPGM-v002) (adenocarcinoma). DNA amplifications and rearrangements were detected by means of FISH (adenocarcinoma and squamous cell carcinoma)

^b^PD-L1 expression was measured by certified pathologists on at least 100 tumor cells with 22C3 antibodies

^c^PD-L1 could not be evaluated in 23 patients as biopsied material was of insufficient quality or quantity

^d^Revised Response Evaluation Criteria In Solid Tumor v1.1, Non evaluable was due to early death of the patient

^e^Durable response was defined as SD, PR or CR for at least 6 months. Those who had a shorter tumor response duration had more often CTC (*p* = 0.01)

**Table 2:** Circulating tumor cells and tumor derived extracellular vesicles

Biomarker	Descriptive	Median (range)/number of patients (%)
CTC at T0 (n = 104)	Median (range)	0 (0–141)
	Patients with CTC	33 (32)
	Patients with CTC > 5	10 (10)
CTC at T1 (*n* = 63)	Median (range)	0 (0–85)
	Patients with CTC	17 (27)
	Patients with CTC > 5	2 (3)
Change in CTC (between T0 and T1) (n = 63)	Median (range)	0 (− 8 − + 39)
	Pts with decrease	11 (16)
	Pts with increase	11 (17)
	Pts with no change	41 (65)
tdEV at T0 (n = 104)	Median (range)	6.5 (0–1753)
	Pts with tdEV≥18	27 (26)
tdEV at T1 (n = 63)	Median (range)	5 (0–1975)
	Pts with tdEV≥18	11 (17)
Change in tdEV (between T0 and T1) (n = 63)	Median (range)	-1 (−46 − + 222)
	Pts with decrease	33 (52)
	Pts with increase	29 (46)
	Pts with no change	1 (2)

Circulating tumor cell (CTC) and tumor derived extracellular vesicle (tdEV) count measured by CellSearch in 7.5 mL of blood aided by automated imaging. For automated imaging the Accept program was used, an open source program introduced by Zeune et al. [20–22]

PD-L1 expression could not be determined in 23 patients (22%) as the tumor material was of insufficient quality or quantity for PD-L1 analysis. From the remaining 81 patients, 44 (54%) had no PD-L1 expression (< 1%), 19 (23%) had PD-L1 expression between 1 and 49% and 18 (22%) had PD-L1 expression ≥50% (Table 1).

Patients with PD-L1 > 50% responded in 9/18 (50%) cases, significantly higher than patients with lower PD-L1 expression wo responded in 17/63 (27%) cases (OR = 3.0, *p* = 0.06 for early tumor response and OR = 2.9, *p* = 0.05 for durable tumor response).

### Presence of CTC

CTC were present in 33/104 T0 samples (32%), of whom most had 1 CTC (*n* = 11/104; 11%). Ten out of all 104 patients (10%) had more than 5 CTC detected. At T1, 17/63 patients (27%) had CTC; of these patients 8 (47%) did not have CTC at T0. Six patients who did have CTC at T0 had no CTC detected at T1. Patients with CTC at both time points showed an increase in three cases, and a decrease in five cases. One patient had 1 CTC per 7.5 mL blood at both time points.

Out of the four patients who had a complete response, 3 had 0 CTC at both T0 and T1. Of the 10 patients with CTC > 5 at T0, two patients had a tumor response (PR), with durable responses being observed in three patients (the two aforementioned patients and one patient with SD).

### CTC and early tumor response

**Fig. 1:**
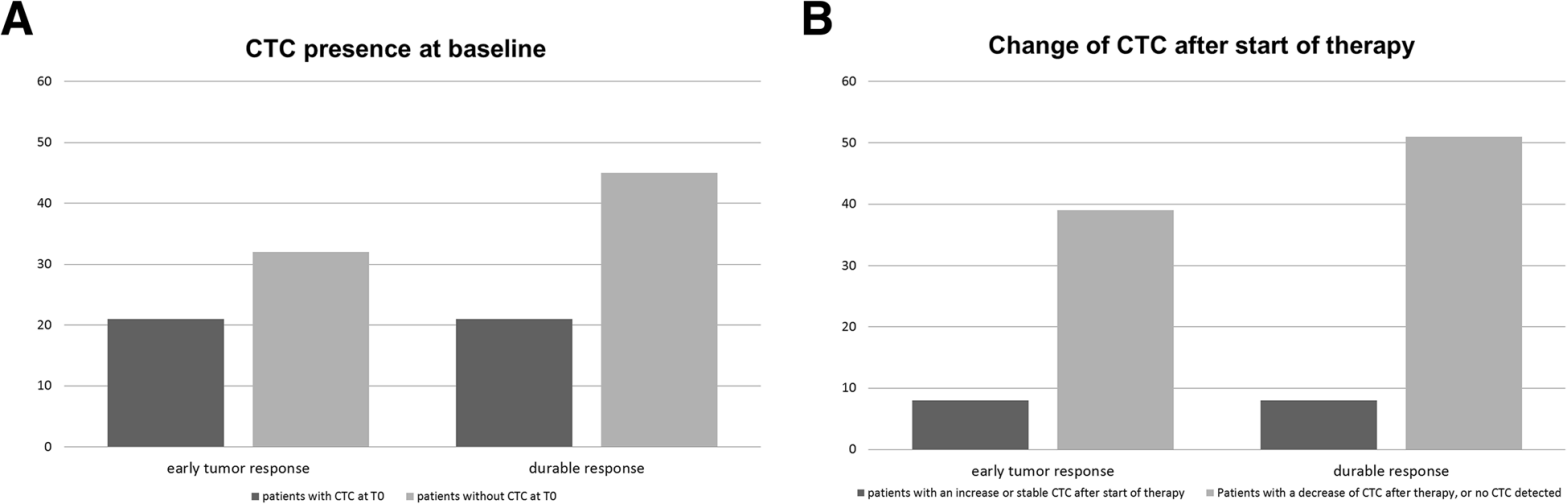
Percentage of advanced non-small cell lung cancer (NSCLC) patients with an early response (partial and complete response according to the revised response evaluation criteria in solid tumors v1.1 [RECIST 1.1],) and durable response (stable disease, partial response and complete response according to RECIST 1.1 without progression in 6 months) to checkpoint inhibitors with and without circulating tumor cells (CTC) at T0 (**a**) and by increased or stable (ΔCTC) CTC counts when measured at 4 to 6 weeks of therapy (**b**). Early response rates were not significantly different (T0: OR = 0.67, *p* = 0.56; ΔCTC OR = 0.13, *p* = 0.08) but durable response rate was significantly lowered in patients with CTC (T0 OR = 0.28, p = 0.02; ΔCTC OR = 0.04, p = 0.01)

Patients with CTC at T0 did not respond differently from those without CTC, with 7/33 (21%) versus 23/71(32%) responding respectively (*p* = 0.2, Fig. 1). Patients with CTC at T1 less often had a tumor response (2/17, 12%) compared to those without CTC at T1 (19/46, 41%; *p* = 0.04), but this difference was not significant after adjustment for other factors (PD-L1, PS, number of organs with metastases and histological subtype) (OR = 0.22, *p* = 0.08).

Patients who had no CTC at either time point (*n* = 40) or decreasing CTC counts (*n* = 11) had a tumor response in 20/51 cases (39%) while patients with increased or stable CTC counts at T1 only responded in 1/12 cases ((8%; *p* = 0.04). In the multivariable analysis this difference in response was no longer significant (OR = 0.13, *p* = 0.08).

### CTC and durable response

Patients with CTC at T0 had a durable response in 7/33 cases (21%), which was significantly lower compared to patients without CTC at T0, who responded in 33/71 patients (46%; *p* = 0.03). This relation remained significant after adjustment for covariables (age, PS, histological subtype, PD-L1, number of organs with metastases, OR = 0.28, *p* = 0.02).

The presence of CTC at T1 was also predictive for lower durable response rates. Patients with CTC at T1 had a durable response in 1/12 cases (12%) compared to 25/46 cases (54%) without CTC at T1 (*p* < 0.01), which remained significant in the multivariable analysis (OR = 0.07, p < 0.01).

Patients with either no CTC at both time points or decreasing CTC, had a durable response in 25/51 cases (51%) versus 1/12 cases (8%) with increased CTC (*p* < 0.01). This association remained significant after adjustment for the selected factors (OR = 0.04, *p* = 0.01).

### Association of CTC with PFS and OS

**Fig. 2:**
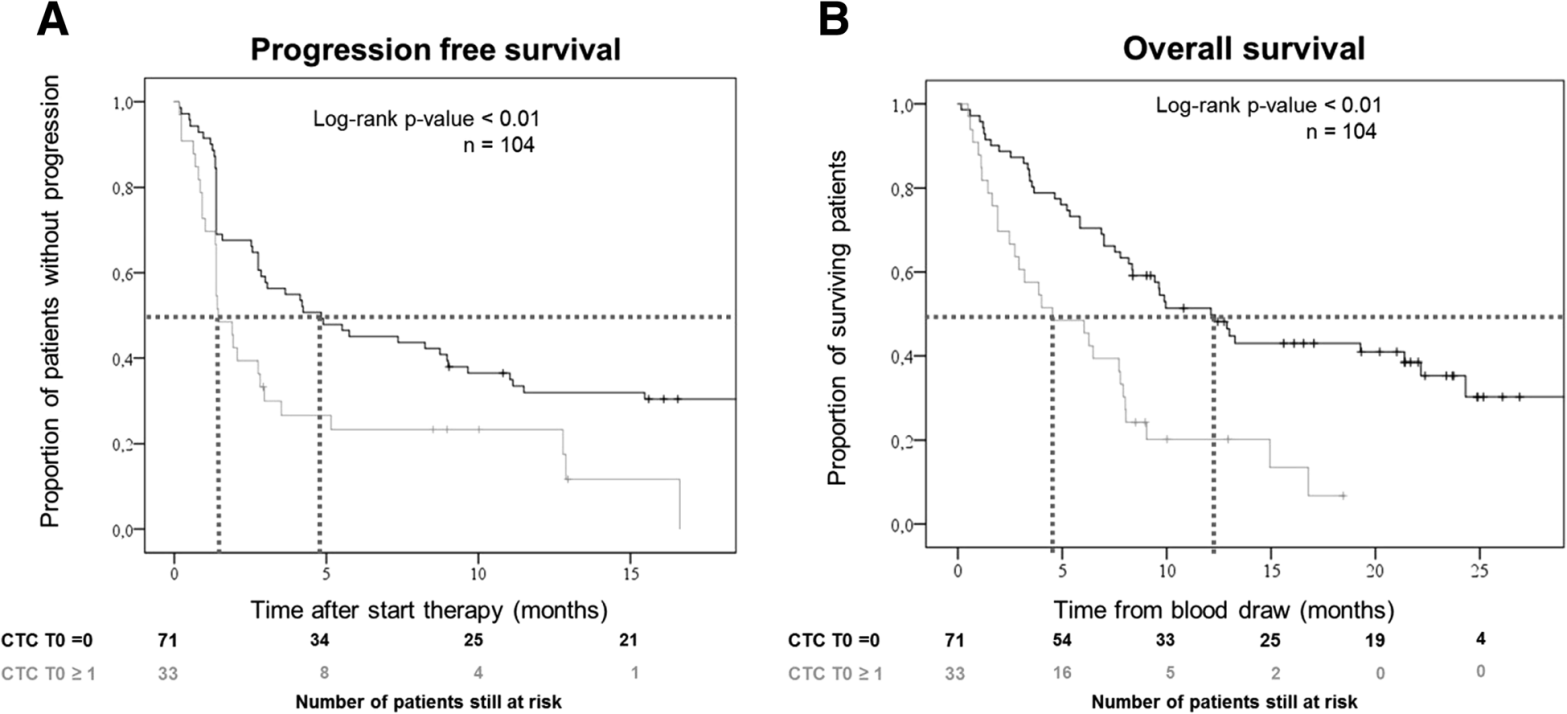
Progression-free survival (PFS, **a**) and overall survival (OS, **b**) of patients with advanced non-small cell lung cancer (NSCLC) treated with checkpoint inhibitors, stratified for baseline circulating tumor cells (CTC). Median PFS and OS of patients with baseline CTC was significantly shorter than that of patients without CTC (PFS: 1.4 months versus 4.8 months, log rank *p* < 0.01, OS: 4.5 months versus 12.1 months, log rank *p* < 0.01)

Presence of CTC, adjusted for PS and histological subtype, was correlated with PFS and OS at T0 (HR = 1.6, *p* = 0.05; HR = 2.2, *p* < 0.01 respectively, Fig. 2), T1 (HR = 3.2, p < 0.01; HR = 3.2, p < 0.01 respectively). An increase in CTC also corresponded with shorter PFS and OS (increased CTC HR = 3.4, *p* < 0.01; HR = 3.7, p < 0.01 respectively).

After adding tumor response as a dichotomous variable to the multivariable model, CTC were no longer significantly correlated to a worse PFS at T0 (HR = 1.5, *p* = 0.13), but remained associated with a worse OS at T0 (HR = 1.89, *p* = 0.02), and worse PFS and OS at T1 (PFS HR = 3.6, *p* < 0.01; OS HR = 2.2, *p* = 0.03) and when CTC counts increased after therapy (PFS HR = 4.46, p < 0.01; OS HR = 2.4, *p* = 0.04).

### Presence of tumor derived extracellular vesicles (tdEV)

At T0, tdEV were present in 94 patients (90%, median 7, range 0–1752), and at T1 in 66 patients (94%, median 5, range 0–1975). There were 26 patients (25%) at T0 who had tdEV≥18, and 10 patients (16%) at T1. In 33 patients (52%) there was a decrease of tdEV while in 29 cases (46%) there was an increase, with only one patient having the same number of tdEV at both measurements (tdEV = 4).

### Tumor derived extracellular vesicles (tdEV) and early tumor response

Patients with tdEV< 18 and tdEV≥18 did not respond differently, with respectively 7/27 (26%) and 23/77 (30%) early responders at T0 (*p* = 0.70, Additional file 1: Fig. S1), and 18/52 (35%) and 3/11 (27%) early responders at T1 (*p* = 0.64).

### Tumor derived extracellular vesicles (tdEV) and durable tumor response

No significant difference in durable response rate was observed between patients with tdEV< 18 and patients with tdEV≥18 at both time points. At T0, 8/27 patients (30%) with tdEV< 18 and 31/77 patients (40%) with tdEV≥18 had a durable response (*p* = 0.33). At T1, 24/52 patients (46%) with tdEV< 18 and 3/11 patients (27%) with tdEV≥18 had a durable response (*p* = 0.25).

### Association of tdEV with PFS and OS

Patients with elevated tdEV were associated with a shorter PFS (T0: HR = 1.8, *p* = 0.03; T1: HR = 2.5, *p* = 0.02; ΔtdEV: HR = 1.02, *p* < 0.01) and shorter OS (T0:HR = 2.4, p < 0.01, T1: HR = 2.8, p = 0.02; ΔtdEV HR = 1.01, p < 0.01) in a multivariable model corrected for PS, histology, number of organs with metastases and PD-L1 (Additional file 2: Fig. S2).

## Discussion

The currently clinically used biomarker for checkpoint inhibitors is PD-L1 expression, but it is not robust enough to predict therapy response on a per patient basis. Tumor mutational burden likely predicts response as well, but is not (yet) routinely used [4, 27–29].

We investigated the role of CTC and tdEV in patients with advanced NSCLC treated with checkpoint inhibitors in a real life patient population and observed that CTC were an independent predictive factor for durable tumor response rates, even after adjusting for other factors [21, 30, 31]. Durable response rates were twice as high for patients without CTC at baseline compared to patients with CTC (OR = 0.28) and even six times as high for patients with decreased CTC counts after therapy compared to increased CTC counts (response OR = 0.04).

CTC were not associated with early tumor response, and tdEV were not associated with either early tumor or durable tumor response, but were associated with worse progression free and overall survival.

The association of CTC with durable response were more pronounced compared to early tumor response, mostly due to stable diseases which remained stable for a long period of time (no early tumor response converting to durable response), and responders progressing within 6 months. It appears that even patients who have an early tumor response or have a stable tumor can continue to disseminate CTC, but these patients are at a high risk for early tumor progression. Therefore CTC could be a reflection of the metastatic potential and aggressiveness of the tumor as postulated by De Wit et al. and others, and determines how fast the tumor can return after an observed tumor response [10, 13, 17]. Another possibility is that CTC may undergo endothelial to mesenchymal transition (EMT), inducing increased expression of genes related to resistance to chemotherapy, which are also seen in possible cancer stem cells [32–35]. Vesicles also continue to be disseminated from patients with early tumor responses, possibly due to apoptosis of tumor cells.

Unfortunately, the clinical applicability of CTC in advanced NSCLC is limited by the low number of CTC that can be found in 7.5 mL of blood. CTC are only observed in around 30% of patients and their absence could be due to the low volume of blood screened, explaining their high specificity but low sensitivity. Methods to yield higher numbers of CTC are being developed, for example by exploring larger blood volumes such as is observed with diagnostic leukapheresis [36–39]. Additionally, when more CTC are available functional analysis can be performed, which could further improve predictive values.

Despite the low detection rate, the presence of CTC, when detected, has clinical implications for survival and response rates. As it is a marker of decreased response when detected, the low detection rate is less of a concern. If our results are confirmed in a larger cohort, CTC could be useful for monitoring disease, allowing for early cessation of treatment with checkpoint inhibitors, omitting CT scans and preventing patients being treated with inferior and aggressive treatment at the end of life.

It is known that CTC are related with survival in NSCLC and several studies have shown that the presence of CTC are predictive of worse tumor response to chemotherapy and targeted therapies [6, 9, 11, 15, 16, 40–46]. CTC in advanced NSCLC are not a homogeneous population. PD-L1 expressing tumors can shed PD-L1+ CTC and these cells are associated with a lower tumor response to checkpoint inhibitors when measured at start of therapy and after 3 months [47–49]. In one study, it was found that patients who had PD-L1 negative CTC 6 months after the start of checkpoint inhibitors benefitted from immunotherapy in most cases, while patients who had PD-L1 positive CTC at that time all progressed. These studies show that subtyping of CTC is possible but their meaning without correction for clinical factors is not known and warrant further analysis.

## Conclusion

We observed CTC in one third of advanced NSCLC patients, who on the long term respond worse towards checkpoint inhibitors. This provides an additional tool for the prediction of checkpoint inhibitor responsiveness, which may be of particular interest for patients in whom no tumor tissue is available for other predictive analysis.

## Funding

The authors participate in the Cancer-ID consortium which has received support from the Innovative Medicines Initiative (IMI) Joint Undertaking under grant agreement No 115749. Its resources are composed of financial contribution from the European Union’s Seventh Framework Program (FP7/2007–2013) and EFPIA companies’ in-kind contribution. The funding source had no involvement in study design, collection, analysis or interpretation of the data or in the writing and submission of the report.

## Additional files

Additional file 1: **Figure S1.** Percentage or early and durable responders by tdEV count ≥18 at baseline. Percentage of advanced non-small cell lung cancer (NSCLC) patients with an early response (A: partial and complete response according to the revised response evaluation criteria in solid tumors v1.1 [RECIST 1.1]) and durable response (B: stable disease, partial response and complete response according to RECIST 1.1 without progression in 6 months) to checkpoint inhibitors with tumor derived extracellular vesicles (tdEV) ≤17 and ≥ 18 at baseline. Response rates were not significantly different between groups (early response OR = 0.89, *p* = 0.58, durable response OR = 0.67, *p* = 0.46). (DOCX 30 kb)

Additional file 2: **Figure S2.** Progression-free and overall survival of NSCLC patients treated with checkpoint inhibitors by baseline tdEV ≥18. Progression-free survival (PFS [A]) and overall survival (OS [B]) of patients with advanced non-small cell lung cancer (NSCLC) treated with checkpoint inhibitors, stratified for tumor derived extracellular vesicels (tdEV) count of at least 18 and higher at baseline (tdEV≥18). Median OS of patients with tdEV≥18 was significantly shorter than that of patients with tdEV< 18 (OS: 3.8 months versus 9.7 months, log rank *p* = 0.02). PFS was significantly shorter for patients with tdEV≥18 in the multivariable Cox regression analysis (median PFS 1.38 versus 4.1, log rank = 0.07, HR = 1.8, *p* = 0.03) (DOCX 127 kb)

## Data Availability

The datasets used and/or analysed during the current study are available from the corresponding author on reasonable request.

## Abbreviations

CTC: Circulating tumor cells
EMT: Endothelial to mesenchymal transition
EpCAM: Epithelial cell adhesion molecule
FISH: Fluorescense in situ hybridization
HR: Hazard ratio
NSCLC: Non-small cell lung cancer
OR: Odds ratio
OS: Overall survival
PD-L1: Programmed death ligand 1
PFS: Progression free survival
RECISTv1.1: Response evaluation criteria in solid tumors version 1.1
tdEV: tumor derived extracellular vesicles
