# Supplementary figures and images for: Circulating tumor cells in advanced non-small cell lung cancer patients are associated with worse tumor response to checkpoint inhibitors
Source: J Immunother Cancer. 2019 Jul 10;7:173. doi: 10.1186/s40425-019-0649-2 (PMC6617698; doi:10.1186/s40425-019-0649-2)

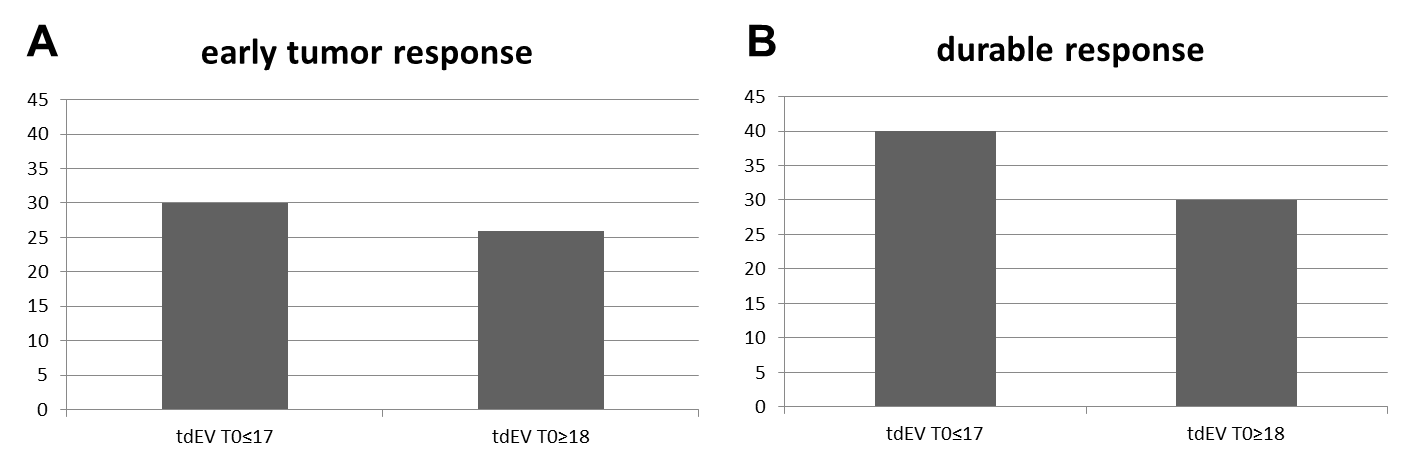

Supplement: Supplementary file 1 — Figure S1. Percentage or early and durable responders by tdEV count ≥18 at baseline. Percentage of advanced non-small cell lung cancer (NSCLC) patients with an early response (A: partial and complete response according to the revised response evaluation criteria in solid tumors v1.1 [RECIST 1.1]) and durable response (B: stable disease, partial response and complete response according to RECIST 1.1 without progression in 6 months) to checkpoint inhibitors with tumor derived extracellular vesicles (tdEV) ≤17 and ≥ 18 at baseline. Response rates were not significantly different between groups (early response OR = 0.89, p = 0.58, durable response OR = 0.67, p = 0.46). (DOCX 30 kb) [file 40425_2019_649_MOESM1_ESM.docx]

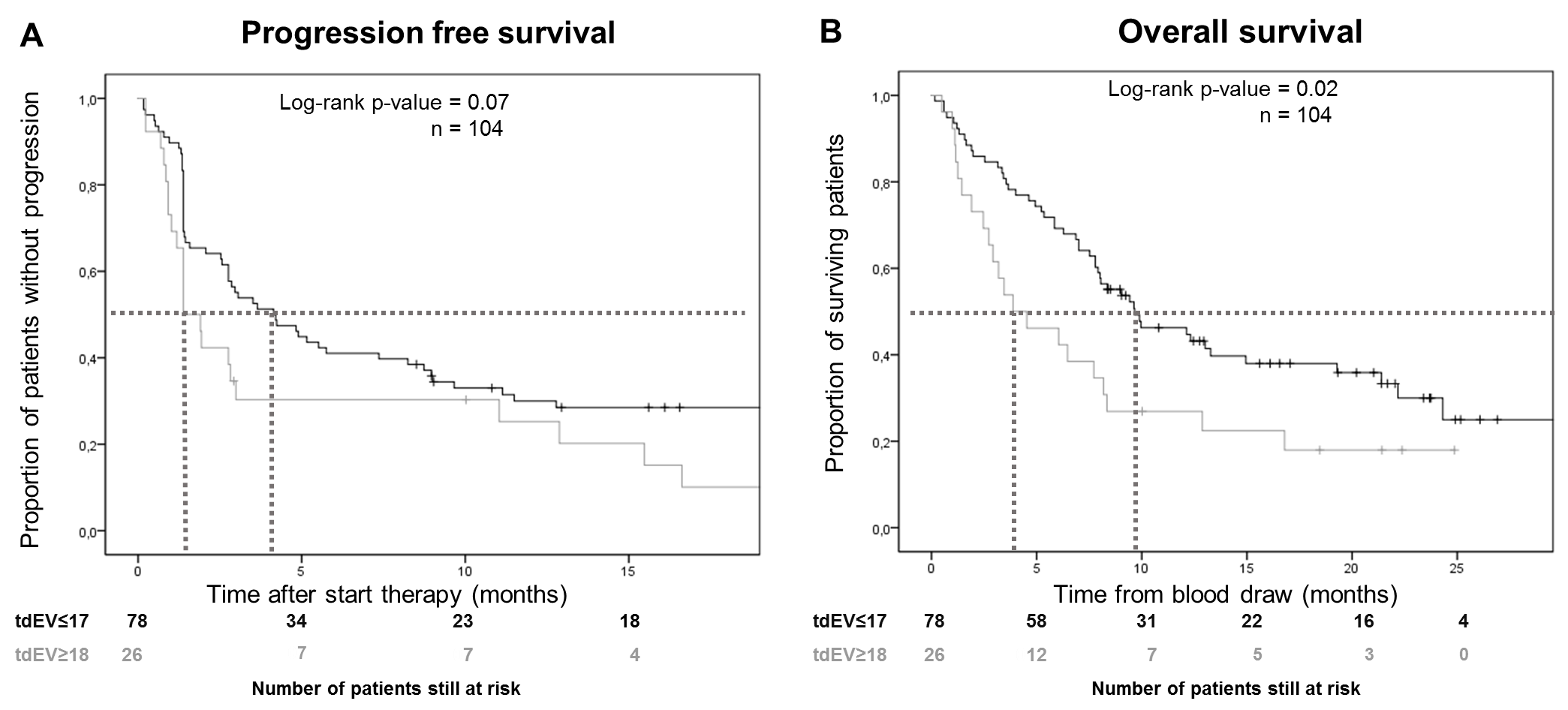

Supplement: Supplementary file 2 — Figure S2. Progression-free and overall survival of NSCLC patients treated with checkpoint inhibitors by baseline tdEV ≥18. Progression-free survival (PFS [A]) and overall survival (OS [B]) of patients with advanced non-small cell lung cancer (NSCLC) treated with checkpoint inhibitors, stratified for tumor derived extracellular vesicels (tdEV) count of at least 18 and higher at baseline (tdEV≥18). Median OS of patients with tdEV≥18 was significantly shorter than that of patients with tdEV< 18 (OS: 3.8 months versus 9.7 months, log rank p = 0.02). PFS was significantly shorter for patients with tdEV≥18 in the multivariable Cox regression analysis (median PFS 1.38 versus 4.1, log rank = 0.07, HR = 1.8, p = 0.03) (DOCX 127 kb) [file 40425_2019_649_MOESM2_ESM.docx]
